# Adopting prospective antimicrobial stewardship (AMS) practice in high-risk immunosuppressed groups: an urgent call to action in the era of antimicrobial resistance (AMR)

**DOI:** 10.1093/jacamr/dlae145

**Published:** 2024-09-27

**Authors:** S Agrawal, A Bapat, J Amos, E Howes, T Ashfield

**Affiliations:** Immunobiology, Blizard Institute, Queen Mary University of London, London, UK; Department of Haemato-Oncology, St Bartholomew's Hospital, London, UK; Department of Infection Sciences, King’s College Hospital NHS Foundation Trust, London, UK; Medical Affairs, Anti-Infectives, Pfizer Ltd, Tadworth, Surrey, UK; Medical Affairs, Anti-Infectives, Pfizer Ltd, Tadworth, Surrey, UK; Medical Affairs, Anti-Infectives, Pfizer Ltd, Tadworth, Surrey, UK

## Abstract

Life-saving immunosuppressive treatments including intensive chemotherapy and bone marrow transplantation expose patients to a considerable risk of death from infection globally. With evolving AMR and transmission, this could spell disaster for patients across the world and society at large. Antimicrobial stewardship (AMS) and prompt appropriate management of potentially fatal, emergent infections are essential. It is now apparent that antibacterial prophylaxis in patients with haematological cancer may not provide survival benefit while simultaneously increasing risks for AMR carriage.

With evolving AMR and increasing immunosuppressed populations across the world, we must institute robust AMS practices. Significant resources are used to combat the impact of AMR on immunosuppressed patients. For lower-middle income countries (LMICs) these resources may not be available and as such the impact caused by AMR is greater. By considering the patient journey holistically we consider risk of infection presented to patients temporally and geographically. A short-term and easy to implement approach of multi-disciplinary team (MDT)-style advance care planning for infection is advocated.

Antimicrobials, when used appropriately, enable healthcare procedures to occur and exist. Indeed, the very future of clinical medicine will rely on this yet to be realized value of enablement. Proactive effort and change must occur across all sectors with holism; hence our impetus for convening a joint industry and clinical working group. With at-risk immunosuppressed groups being a sentinel for change, awareness and implementation of patient-centric actions for infection are essential and our recommendations serve as an urgent call to action.

## Introduction

Antimicrobial resistance (AMR) is an urgent humanitarian threat that is on us; our collective response matters right now.^1^ As microbes become resistant to the treatments available, we are at greater risk. A particularly vulnerable group across the world are the immunosuppressed^2^ for whom antimicrobial stewardship (AMS) practice is paramount.

### What is antimicrobial stewardship (AMS) and what value does it provide?

AMS is a set of coordinated interventions to improve and measure the use of antimicrobials by promoting an optimal drug regimen including dosing, duration of therapy and route of administration.^3^ We must embrace proactivity and holism in AMS by considering patient needs, risk factors, the infection syndrome and the pathogen.

Antimicrobials are the ‘fire extinguishers’ of modern healthcare.^4^ Their use, when well stewarded, provides an enablement to healthcare that underpins our ability to safely perform high-risk interventions such as surgery and haematopoietic stem cell transplantation.^5^ Without effective antimicrobials, the viability of transplant medicine, chemotherapy and invasive procedures is threatened. To avoid the real prospect of a post-antibiotic era, AMS must be a priority for society and global health.

### AMS in high-risk immunocompromised groups

Advance planning allows for intuitive and tailored risk management. A prime example is rectal screening to detect carriage of resistant organisms, which is recommended for patients undergoing high-risk procedures and can guide targeted empirical therapy.^6^ Immunosuppressed patients represent a sentinel for change. AMS and antifungal stewardship for such patients have been adopted due to necessity. Antifungal stewardship is nuanced and has been embraced by non-infection experts, such as haemato-oncologists, taking a multi-disciplinary approach to achieve its aims^7^ as well as serving as an exemplar for AMS domains such as diagnostic stewardship^8^ and the interplay of the surrounding environment.^9^

However, global disparities present resource barriers for these novel approaches in LMICs. Enlightened self-interest allows the better resourced nations to help in the fight, which includes reciprocal sharing of surveillance data and of resistance detection in health tourists.

AMS focussed behavioural change has yielded beneficial outcomes including significantly lower risk of ICU admission and mortality in high-risk neutropenic patients.^10^ An added benefit in this study was a reduction in carbapenem and glycopeptide use, both of which are classified as either watch or reserve antibiotics within the WHO AWaRe classification.

Patients undergoing specialized treatment are likely to pass through multiple healthcare environments that expose them to risk. Proactive care planning for AMS can be instituted in any setting to a greater or lesser extent. Risk assessment serves as the baseline and should consider a personal history of antimicrobial consumption, foreign travel, admission to healthcare settings with increased AMR prevalence and an appreciation of the epidemiology of the community and healthcare metabiome.

An increasingly relevant consideration is gut microbiome health. With antimicrobial exposure, a vicious cycle of selection for more resistant bacteria and complex infection can result. Indeed, microbiome signatures can predict infection risk in patients receiving induction therapy for acute myeloid leukaemia^11^ and gut translocation of pathogenic organisms is known to cause invasive disease.^12^ Additionally, even in the absence of antimicrobials, the human gut microbiome can function as a reservoir for resistance genes and amplify AMR.^13^

Solid organ transplant (SOT) recipients also face a significantly higher risk of severe infection and mortality.^14^ Infection in SOT recipients is uniquely challenging due to the specific organs affected and chemotherapies used. For example, enteric Gram-negative bacteria and *Candida* are commonly known to cause morbidity in post-liver transplant recipients while invasive pulmonary aspergillosis is a feared complication in post-lung transplant recipients.^15^

While there can be no ‘one-size-fits-all’ AMS approach for SOT recipients, common themes essential for successful AMS development include: engagement from transplant physicians in the creation of SOT-specific AMS teams, collection of local AMR rates and infection patterns and the creation of SOT-specific antibacterial and antifungal prescribing policies.^16^

### An illustrative case: AMS practice yielding favourable AMR outcomes in high-risk immunosuppressed groups

An example of this paradigm approach is characterized by Caldwell *et al.*^17^ who explored the impact of fluoroquinolone prophylaxis in stem cell transplantation and intensive chemotherapy. Routine antibacterial prophylaxis was discontinued due to concerns over increasing AMR and lack of efficacy.

The proportion who survived at 7 and 30 days post-bacteraemia was similar between the groups that received prophylaxis or not (*P* = 0.61). However, there was a significantly lower rate of ciprofloxacin-resistant *E. coli* bacteraemia [73% versus 29% (*P* = 0.001)] and ESBL resistance in those that did not receive prophylaxis.

In the follow-on study, 2391 high-risk haemato-oncology admissions were analysed over a 5-year period before and after cessation of ciprofloxacin prophylaxis.^18^ A higher rate of emergent Gram-negative blood stream infection was detected, but with a lower AMR risk. A significantly lower mortality amongst patients who did not receive prophylaxis was noted. These findings tempt us to question blanket prophylaxis policies that may, in fact, be a cause of harm, and incites a need to undertake prospective care planning for cohorts and individuals.

## Recommendations and summary

We have described the contemporaneous and evolving threat of AMR and the relevance of AMS for high-risk groups. As we encounter an increasingly immunosuppressed world, the implementation of AMS must be at the forefront of practice and policy, and as such these groups act as a sentinel for change. The featured study illustrates an ability to adapt to local epidemiology, while maintaining prompt infection management of incipient infection.

Our collective responsibility for optimized infection practice and AMS is of vital importance. We require vigilance, surveillance, communication and applied logistics to meet such aims and adapt to an evolving threat. Optimizing pathways and AMS are equally important for other patient cohorts; in this vein, AMS strategies for high-risk patients can inform approaches for all, not just the immunosuppressed.

To meet this challenge, we call for collaborative actions at individual practitioner level, within health care systems and within policy (see Table 1). This extends to all sectors including industry, charities, patient groups and healthcare agencies. Applying diagnostic, therapeutic and technological solutions should complement these ambitions.

**Table 1.** A call to action for AMS focussed actions in the battle against AMR for individuals, systems, and policy with a focus on high-risk patient groups

Individuals	Healthcare systems	Policy and technologies
Practitioners Practice of AMS and infection control as a central priority (each antimicrobial prescription has a public health impact) Be responsive to local epidemiology and practice change Audit own antimicrobial and AMS practice Maintain education and awareness of AMR and AMS Awareness of high-risk procedures and their impact on infection risk Embrace advance care planning approaches for infection Awareness of microbiome effects from antimicrobial use Patients Patient awareness of AMS and AMR, empowered learning Understanding of immunosuppression Patient awareness of pathways for clinical care Carer support Patient information provision on AMR, antimicrobial history, admissions and travel Infection prevention messaging	Pathways Establish infection specific pathways applicable cross-specialty Trusts Board-level representation for and leadership AMR/AMS Adaptive dynamic policies responsive to changes in epidemiology Reduce silos between departments/specialties/laboratories Risk stratified list of high-risk procedures e.g. immunosuppression Primary care/community liaison Embrace advance care planning approaches for infection Regional Board-level representation for and leadership AMR/AMS Incite AMS policies for regional adoption Dynamic and relevant surveillance National Share best practice Enhance existing surveillance systems and communications Institute AMS and infection outcome registries National ranked risk level for high-risk procedures Pandemic preparedness regarding AMR Adapt to novel and pipeline technologies e.g. therapeutic, diagnostic, digital	Policy Mitigate silos between health agencies Improved outcome/burden measures e.g. mortality and morbidity regarding infection and AMR One Health focus to consider environmental surveillance (buildings, wastewater, air quality) AMR National Action Plan adherence Global relations Public awareness and societal value of AMR and AMS Laboratory Cohesive dialogue from laboratory to patient Data to inform regional and national surveillance Screening, surveillance and trends Data approaches that can inform local antibiogram approaches Logistic solutions that resolve time to definitive diagnosis Technologies Invested research and networks related to AMS/AMR Maintaining an innovative antimicrobial pipeline Maintaining appropriate diagnostics including near patient testing Attendance to national and global stewardship frameworks

The environmental metabiome and human microbiome are now recognized as key factors for AMR and worthy of mention for a future holistic and robust system. For example, screening the microbial composition of wastewater from healthcare facilities may play a putative role in decision-making.^19^

Infection management transcends all systems, specialities and patients. Furthermore, each antimicrobial prescription has an impact on population risk for AMR and, in this context, these medicines have been referred to as societal drugs.^5^ Given that infections occur in every aspect of healthcare, it is neither desirable nor achievable to have specialist AMS input for all infections. Realizing the true enablement value of good infection management and AMS provides strong rationale for this practice to be embraced by all. To achieve this, there needs to be an uplift in the value given to infection specialties.

In certain clinical scenarios, the necessity for urgent empirical treatment means there will always be a time lag time to a definitive infection diagnosis and hence the possibility of exposure to inappropriate antimicrobial use. Reducing this lag time with a more holistic approach and adept diagnostic pathway remains the holy grail to optimal management.

Appropriate surveillance data and outcome registries are likely to manifest in time and permit some of the aspirations recommended here. Idealistically, such data should be available in close to real-time. In this regard, infection management approaches could be dynamic and adapt to present changes in epidemiology.

LMICs face different challenges and in some cases far higher rates of AMR. Our principle of advance care planning requires few resources and relies predominantly on taught knowledge so can be translated to such areas even with lack of resource and by instituting a stewardship mindset within clinical teams. There are strong examples of leadership and change in such areas and resources exist to support the implementation of national action plans.^20^

By appreciating the time-critical nature of AMR through a lens of high-risk immunosuppressed groups, we can realize care gaps and the areas of need to be addressed. Our recommendations are ostensibly holistic and aspirational but within reach. We provide framework recommendations for whole system change with calls to action for the individual, systems, and policy. Only with such aspiration and holism can we affect the ongoing evolution of AMR and meet the needs of all patients.

## Transparency declarations

S.A.—advisory boards, research and educational grants with Gilead, Pfizer, Shionogi, MundiPharma/NAPP. A.B.—Napp/Mundipharma, Gilead. J.A.—Previous employee and shareholder of Pfizer Ltd, patient engagement and public safety committee member of Antibiotic Research UK. E.H.— previous employee of Pfizer Ltd. T.A.— employee and shareholder of Pfizer Ltd, at the time of writing, council member of British Society for Antimicrobial Chemotherapy, GARDP antimicrobial Chemotherapy Conference 2025 Scientific Committee, recent consultancy contracts with Menarini, ADVANZ, FluoretiQ, ESSITY and Binary Pharma.
